# A quantitative appraisal of selected agroforestry studies in the Sub-Saharan Africa

**DOI:** 10.1016/j.heliyon.2022.e10670

**Published:** 2022-09-17

**Authors:** Kennedy Muthee, Lalisa Duguma, Christine Majale, Monicah Mucheru-Muna, Priscilla Wainaina, Peter Minang

**Affiliations:** a World Agroforestry (ICRAF), UN Avenue Gigiri, Nairobi, Kenya; b Department of Spatial and Environmental Planning, Kenyatta University, Nairobi, Kenya; c Department of Environmental Sciences and Education, Kenyatta University, Nairobi, Kenya; d Global Evergreening Alliance (GEA), 12/24 Lakeside Drive, East Burwood VIC 3151, Australia

**Keywords:** Agroforestry, Climate change, Ecosystem services, Livelihoods, Nature-based solutions, Sub-Saharan Africa, Systematic review

## Abstract

The multiple ecosystem services and livelihood assets development challenges facing the world, including climate change, land degradation, and high poverty levels, have necessitated cross-cutting solutions. Such includes agroforestry technologies, where trees are integrated with crop and pasture lands to yield multiple ecosystem goods and services. Though an ancient approach to land management, agroforestry faces a modern and urgent demand for expansion to counter ecosystems-livelihoods imbalances in most regions across the globe. This paper sought to synthesize the dynamics and characteristics of agroforestry technologies in sub-Saharan Africa by adopting the systematic review approach. Eighty-six (86) agroforestry studies were reviewed, analysing variables such as the dominant agroforestry technologies, production systems, types of studies, and ecosystem services generated by different agroforestry technologies. It established that majority of the agroforestry studies are multiple (undefined) in nature at 36%, have moderately changed over the years, the dominant agroforestry study type is journal articles (59%), and they are mostly scientific in nature (57%). Further, income generation was the dominant provisioning service (31%), greenhouse gas emission reduction was the main regulatory service (31%), and soil fertility management was the key support service. Tradeoffs associated with agroforestry technologies, including increased deforestation rates, tree-crops competition, increased pests and diseases, and potential food insecurity due to reduced crop production were also identified. Barriers to agroforestry such as insecure land tenure systems and inadequate research development are discussed. Pathways towards increased agroforestry technologies adoption, such as creating a conducive institutional and policy environment, as well as developing business support services for agroforestry-related goods and services were identified. The study reiterates the need for increased agroforestry technologies adoption to create the ecosystems-livelihoods balances, with sufficient measures to minimize the potential tradeoffs.

## 1 Introduction

The sub-Saharan Africa (SSA) region continues to grapple with climate variability and change effects, with the vast of its population depending on its immediate ecosystems for survival. As a livelihood practice, agriculture is the dominant economic activity in the region, with Niang et al. (2014) estimating its increase by 57%, consequently leading to a 16% decrease in forest cover and a 15% increase in barren land between years 1975 and 2000. However, challenges such as decreasing land fertility, reduced per capita land holding, climate change effects, among others, are continuously constraining this practice (Bjornlund et al., 2020), which has necessitated cross-cutting and nature-based solutions that can yield multiple livelihoods and ecosystem services benefits.

Though an ancient land management practice dating back to the middle ages (King, 1987), most of the deliberate research, policy and developments that characterize the modern agroforestry concept came of age in the 1970s as Nair (1993) and van Noordwijk (2019) establish. The point of convergence on the definitions fronted by different studies is that agroforestry is a deliberate integration of trees in a particular temporal or spatial sequence in agricultural or pastoral lands including forests and forest margins farming, to yield multiple ecosystem services and livelihoods benefits. For over half a century now, scientists across the globe have explored various angles and dimensions in which agroforestry is practised, potential benefits and challenges constraining its full adoption (King, 1987; Hsiung et al., 1995; Bjornlund et al., 2020; Kumar et al., 2020). Trends and patterns have significantly moved from simple ‘plot and farm level’ agroforestry technologies that farmers with minimal scientific backing locally develop, to the modern ‘landscapes, ecosystems and livelihoods level’ agroforestry technologies that are techno-scientifically developed and characterized by complex vegetation and components structures (Foresta et al., 2000; Kumar et al., 2020; van Noordwijk, 2019). In their final forms, complex agroforests have ecological functioning and ecosystem services provision that mimics that of natural forests (Foresta et al., 2000; Asase and Tetteh, 2010). Nair et al. (2009) estimated that 1,023 million ha of land globally is under agroforestry, a number that is likely to have increased over time.

Achieving the ecosystem services and livelihood security balance with minimal tradeoffs remain a major challenge facing many practices and concepts, including agroforestry. Well-designed agroforestry technologies should meet both ecosystems and livelihood needs. Smith (2010) points out some of the potential ecosystem benefits including enhanced biodiversity conservation, water quality regulation, soil and air quality, and control of pests and diseases, while simultaneous livelihood needs may include income generation, economic stabilization, food security, and livelihoods diversification. In some cases, however, agroforestry technologies have generated more tradeoffs than synergies between ecosystem services and livelihood securities, including crop-trees competition for available resources leading to decreased farm productivity as Vaast and Somarriba (2014) note. Previous studies have already established the need for a ‘system-wide approach’ as opposed to a *‘*sectoral approach’ in resource planning and use to minimize the potential tradeoffs (see for example: Muthee et al., 2017, Muthee et al., 2021; Zinngrebe et al., 2020; Duguma et al., 2021; van Noordwijk, 2021). This study posited that agroforestry reduces tradeoffs and maximizes synergies between ecosystem services and livelihoods security if well developed, especially in the SSA region. The study employed a systematic review approach on the trends and patterns of agroforestry studies in the SSA region to understand how the duality of ecosystem services and livelihoods security has been framed and areas of synergies and tradeoffs in this framing.

## 2 Eras of agroforestry research and practice development

### 2.1 The era of traditional agroforestry technologies—‘trees on plots and farm level’ (before 1970s)

Agroforestry is an ancient land use and management technology. The period before 1970 is largely viewed as an era of agroforestry conceptualization, which was dominated by traditional practices. King (1987) established that communities globally adopted traditional practices of cultivating trees and crops, including shift cultivation, slash and burn, and forest farming since the Middle Ages (roughly between the 5th and 15th centuries). Such technologies were common across Europe, with some parts of Germany practising it until the 1920s (King, 1987). In parts of Asia, valuable trees were either planted or spared during agricultural intensification to preserve their value to agroecosystems and livelihoods in the Hanunoo farming practices (Conklin, 1957). In China, forest burning for crop production through nomadic slash and burn was recorded as a dominant farming practice in the New Stone Age period (7000-8000 BC), which evolved into settlement farming in Han Dynasty period (2000-1600 BC) according to Hsiung et al. (1995).

In the tropical America, integrating crops and trees in different layers was common, while in the sub-Saharan Africa communities adopted different agroforestry technologies based on the level of knowledge and sociocultural systems (King, 1987). Such technologies included growing food crops under trees to boost productivity and ecological functioning, such as erosion reduction, soil fertilization and provision of shade (Raintree and Warner, 1986). Deliberate agroforestry technologies, largely modified from the traditional practices, started to emerge in the late 19th century, including Taungya which was adopted in Teak plantations in Burma and later South Africa and Shamba system in Kenya in the early 1900s (Nair, 1993). Despite their existence, very few of these agroforestry technologies had been critically studied and analysed from a scientific research point of view (van Noordwijk, 2019), which formed a strong basis for the subsequent era that delved deeply into the development and institutionalization of agroforestry.

### 2.2 The eras of agroforestry concept development and institutionalization**—**‘agroforestry at landscapes and livelihoods scales’ (the 1970s–1990s)

In the book edited by Steppler and Nair (1987), 1977–1987 is described as the ‘decade of agroforestry development’. The era saw the proper definition of the term agroforestry in 1977 as a *‘land use practice where trees are grown on farms in a spatial, temporal or rotation arrangements’* (Lundgren, 1982) to achieve ecological and economic benefits to the farmers. Huxley (1983) described agroforestry as a ‘new science’, with many aspects of plant science remaining unknown. Further, the era witnessed increased studies connecting agroforestry theories and practices, role of agroforestry research in development, socioeconomic influencers to agroforestry, and potential innovations (Franzel et al., 2002). However, agroforestry scaling remained a major constraint.

During this period, some of the landmarks included the establishment of the International Council for Research in Agroforestry (World Agroforestry) in 1978, which was a crucial step in institutionalizing agroforestry in Africa and Asia. Concurrently, Germany invested substantial research in agroforestry research development in Latin America between 1970s and 1990s through institutions such as The Tropical Agricultural Research and Higher Education Center (CATIE) (Muschler, 2016). The Sub-Saharan Africa countries also made deliberate efforts to mainstream agroforestry, with Kenya being among the first countries to develop a National Seminar on Agroforestry in 1980 laying a strong ground for agroforestry research development and institutionalization nationally (Buck, 1981), with the second conference in 1988 setting the guidelines and strategies for agroforestry scaling up and intensification in the coming decades (Kilewe et al., 1989). Through United Nations University, the UN also held various strategic meetings in Costa Rica, Thailand, Nigeria, and Germany during this era exploring the socioeconomic and institutionalization of agroforestry (UNU, 1984). The institutionalization process of agroforestry saw its mainstreaming in national laws and policies framework to bridge agriculture and forestry. By the late 1990s, agroforestry concept and institutionalization were at a mature stage with clear principles, which was crucial for agroforestry scaling up and intensification globally as Huxley (1999) notes in his book *Tropical Agroforestry*. The book is among the first analytical account of the principles and practices of agroforestry as they apply to sustainable land management.

### 2.3 The era of agroforestry research and policy mainstreaming**—**‘governance, policy and science aspects of agroforestry’ (the late 1990s to date)

**Figure 1:**
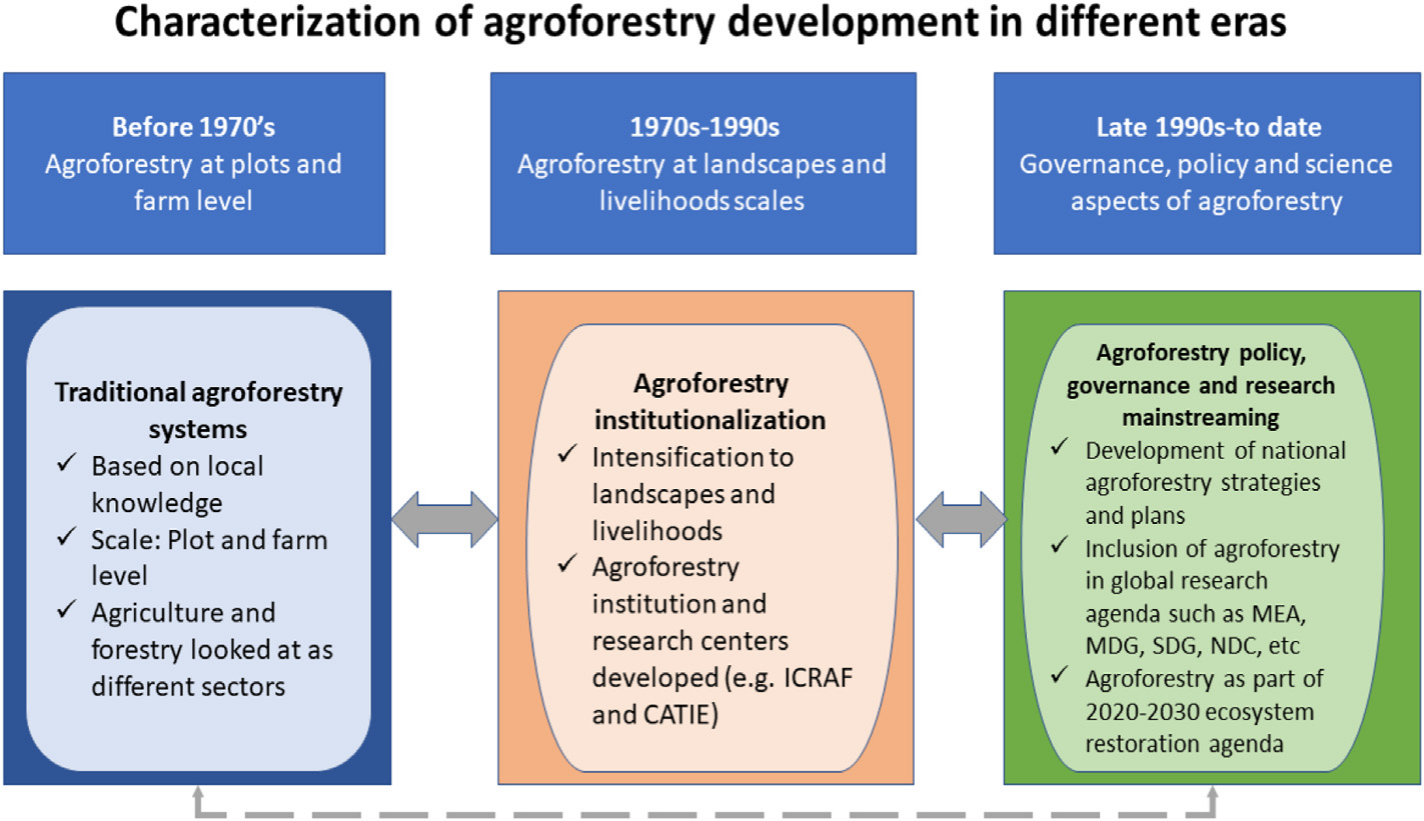
Characterization of agroforestry development in different eras (Source: Author). Note: Some of the key progresses in different eras of agroforestry development connection between the traditional and modern progress in agroforestry research. Its notable that some rural communities still practice still practice the traditional agroforestry practices even in these modern eras based on their awareness and capacity levels.

van Noordwijk et al*.* (2019) look back into the five decades of agroforestry development, a journey that has moved from field and farm level, landscapes level, to the current scaling and intensification level as a single land-use system. The current agroforestry technologies are complex, multidisciplinary, and interdependent in nature (Asase and Tetteh, 2010; Luedeling et al., 2014), developed to meet multiple ecological and livelihood benefits (Sollen-Norrlin et al., 2020). They are shaped at various scales from farm to global level and mainstreamed to meet the local and national context needs and simultaneously feed into global processes. van Noordwijk et al*.* (2019) argue that there is a move toward increased synergies between forestry and agriculture through agroforestry with minimal tradeoffs. To illustrate, studies by Duguma et al. (2017) and Rosenstock et al. (2019) establish that agroforestry is cited in over 85% and 40% of selected Nationally Determined Contributions (NDCs) respectively mainly within sub-Saharan Africa, as a pathway to meet their conditional and unconditional commitments to greenhouse gas emissions reduction. This can be achieved through increased carbon sequestration potential, avoided deforestation, and avoided degradation using agroforestry practices. Studies such as Waldron et al. (2017) and Andersson (2018) explore the contributions of agroforestry to sustainable development goals (SDGs), with strong impact potential associated with SDG 1 (towards poverty reduction), SDG 2 (alleviating hunger), SDG 15 (life on land) and SDG 13 (climate action). Agroforestry is also covered extensively in the agriculture sections of country-specific National Adaptation Plans (NAPs), with the word ‘agroforestry’ appearing in about two-thirds of the total NAPs (Meybeck et al., 2020). The declaration of 2021–2030 as the UN decade of ecosystem restoration provides a great platform going forward, where countries and regions can put together concerted efforts to protect and rejuvenate degraded ecosystems, more so the agroecosystems that have continuously faced excessive degradation to meet global food security. A quick illustration of some of the characteristics of agroforestry development in the three eras is presented in Figure 1.

### 2.4 Significance of agroforestry practices in small-scale farming in different scales and contexts

Most of the agroforestry practices are applicable to farm dwellers who are highly exposed to climate change effects and variability, among other human and natural crises both at regional and global scales. Establishing trees in agricultural systems cushions the practitioners from total losses that may be accelerated by factors such as increasing temperature and erratic rainfall patterns, which are common occurrences in the regions. To illustrate, the adoption of home gardening systems in East Africa has led to a significant increase in vegetable cultivation and consumption to meet nutrition needs, though with a more bias towards commercial vegetables for income generation (Depenbusch et al., 2021). However, higher impacts and adoption of such interventions need closer training, follow-ups, and monitoring to document impacts. Trees on farm can modify the microclimate in dry and exposed lands, which can make a global significance in the era of climate variability and change and its associated negative effects. For example, establishing trees along the farm boundaries or developing alley agroforestry systems aids in slowing wind speed, regulating air temperature and radiation flux, thus creating an ideal condition for photosynthesis and underneath crops and shrubs regeneration (Monteith et al., 1991). Combining *Zizyphus Mauritania* trees and *mungbeans*, for example, has increased the beans’ yield by 20% (Singh et al., 2018). Even during the instances of total crop failure, households with tree systems can still benefit from the sale of timber and non-timber products as an alternative source of livelihood, acting as a safety net for the households.

Agroforestry trees are also critical in improving soil fertility and biological nitrogen-fixing, an ecosystem service that can improve global food production with minimal costs and environmental trade-offs when compared to chemical fertilizers. Leguminous trees such as *Acacia nilotica* and *Acacia albida* are multipurpose in nature and well adapted to the agroclimatic conditions of the sub-Saharan African region. They improve soil structure and fertility, enhance water retention, nutrients recycling, above ground litter, among other processes that contribute to soil fertility that can boost farm crop productivity. Trees have deep root systems that draw nutrients and water from lower to higher soil layers from crop use. A study by Samra and Singh (2000) concluded that combining trees and crops can rehabilitate degraded agroecosystems through increased soil organic carbon. These roles are critical in improving farm productivity and reducing the usage of agrochemical fertilizers that costly to the farmers. By proving an alternative to chemical fertilizers for crops, trees not only aid in supporting soils’ natural regeneration but also reduces household costs related to enhancing farm crop productivity.

It is also noteworthy that on-farm trees provide a shading effect to the livestock that adds to their productivity, especially in sub-Saharan Africa where livestock development faces heat stress as a significant challenge. Trees reduce heat load by providing shade and cooling environment for livestock (Edwards-Callaway et al., 2021), which leads to lower respiration rates, lower body temperature and panting. Consequently, the animals preserve more energy which is diverted towards increasing milk and meat production. In addition, on-farm tree species provide feed and meet the nutritional needs of the animals. According to Franzel et al. (2014), fodder trees such as *Calliandra calothyrsus, Leucaena diversifolia* and *Sesbania sesban* are planted by over 200,000 smallholder farmers with women accounting for between 40%–50% of the number, with an aim to meet the dairy cows’ protein requirement and subsequently increasing milk and meat production and household income generation.

## 3 Materials and methods

### 3.1 Inclusion and exclusion criteria for selected studies

**Figure 2:**
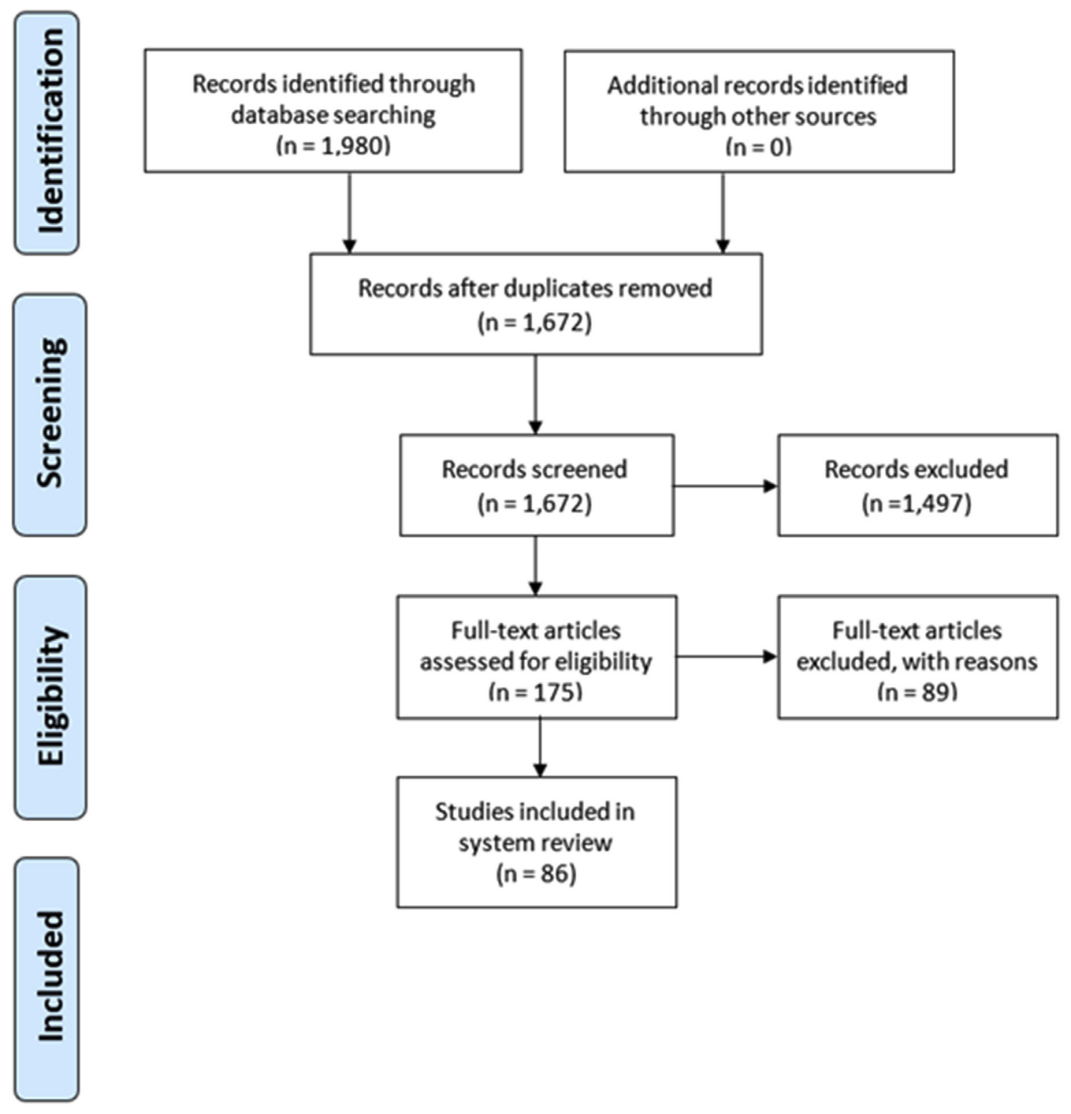
PRISMA guidelines on studies selection.

A systematic review approach was used to retrieve related studies for detailed analysis as employed in studies such as Malkamäki et al. (2018) and Muthee et al. (2022) to identify how the concepts, methods and practices of agroforestry have evolved in the last half a century along the ecosystem services and livelihood security areas. Grey and peer-reviewed literature were sourced from Google Scholar, Scopus and Crossref between 2nd and 3rd August 2021. The Publish or Perish^1^ software was used to search for different phrases and keywords including 'Agroforestry' and 'ecosystem services' and 'livelihoods' and 'tradeoffs' and 'synergies' and 'sub-Saharan Africa'. The retrieved studies were scrutinized through the PRISMA^2^ guidelines as summarized in Figure 2 to establish the studies that met the inclusion and exclusion criteria. The literature selection focused on synergies and tradeoffs in the three disciplines The next phase involved filtering the studies titles and abstracts of the studies to assess their relevance with a final database of 86 studies selected for detailed review and discussion. The studies were categorized per year of publication, county of study within SSA, nature of the study, thematic area of the study and synergies-tradeoffs in ecosystem services and livelihoods in relationship to agroforestry.

## 4 Results and discussions

### 4.1 Agroforestry technologies typologies

The study established that most primary and review studies focused on one agroforestry technology, while most of the conceptual studies were either multiple or undefined in nature (36%). Some of the dominant agroforestry technologies include improved tree fallows, intercropping trees with crops, fodder agroforestry, soil conservation and terracing, as summarized in Table 1. Note that the number of mentioned practices is higher than the reviewed studies since some studies included several practices in their review and analysis.

**Table 1:** Number of mentions of different agroforestry practices in the reviewed studies.

Agroforestry technology	# of mentions	% of the total mentions
Multiple/Undefined systems	36	34
Cocoa agroforestry	10	9
Improved tree fallows	8	7
Parkland agroforestry	7	7
Mixed intercropping	6	6
Homegarden systems	4	4
Fodder shrubs/trees	3	3
Fruit orchard	3	3
Fertility management	3	3
Woodlots	3	3
Coffee agroforestry	3	3
Cacao agroforestry	3	3
Silvopastoral system	3	3
Alley cropping	2	2
Terracing	2	2
Live fencing	1	1
Scattered trees	1	1
Windbreaks	1	1
Relay cropping	1	1
Bioenergy crops	1	1
Multipurpose trees	1	1
Bamboo Agroforestry	1	1
Taungya system	1	1
Tree legume-temperate grass agroforestry	1	1
Cassava based agroforestry	1	1
*Alnus acuminata* based agroforestry	1	1
**Total**	**107**	**100**

#### 4.1.1 Agroforestry technologies characterization

Characterization of the agroforestry technologies was conducted to define the particular practise and its attributes, agroecological niche, different functional roles, and some of their dominant tradeoffs. Different AF technologies synergize crop cultivation within agroecosystems. To illustrate, integrating fertility trees within croplands enhances crop productivity, coffee agroforestry creates a conducive shade for coffee to thrive, while terracing agroforestry aids in soils and water retention for crop growth. Notably, the dominant productive roles of different technologies were the production of food, fodder, and fuelwood; protective functions were dominated by soil and water conservation, while socioeconomic support was provided through income generation and diversification among the practitioners as summarized in Table 2.

**Table 2:** Characterization of dominant agroforestry technologies in the SSA region.

Types of agroforestry technologies	Definition	Agroecological niches	Functional roles			Tradeoffs	Selected references
			Productive	Protective	Social-economic support		
Cassava-based agroforestry	Agroforestry technologies dominated by cassava, intercropping with trees and/or food and cash crops.	Adaptable to various agroecological zones (arid Sahel, highlands and lowlands)	Biofuel feedstock, firewood, human food, animal feed, & industrial starch	Easing pressure forests	Income diversification	Aggravated soil erosion, fertility depletion, topsoil loss, forest loss and degradation	Onoja et al. (2019), Faße et al. (2014), Delaquis et al. (2017)
Taungya system	A shift cultivation agroforestry technology where communities cultivate food crops in forests until the trees mature enough. Dominant taungya types include leased, departmental and village systems.	Adaptable to different highlands and lowland forest types	Food crop production	High tree survival rates, soil fertility	Income to farmers and forestry department, reduced costs of forests plantation development, employment generation	Conflicts in land tenure systems, legal tussles between forestry departments and farmers on land use, tree-crop competition for available resources	Akamani and Holzmueller (2017), Steppler and Nair (1987), Menzies (1988)
Relay and mixed intercropping	Intercropping technology where succeeding crops are planted before harvesting the first crops within agroforestry lands	Adaptable to different ecological zones depending on the crops-trees combination	High food and biomass production, efficient resources use, crop diversification	Increased soil fertility with the right crops-trees rotation, erosion control, pests & diseases control	Increased farm returns and profitability	Soil infertility and acidification due to continuous food production, tree-crop competition for resources e.g., light, water, and nutrients	Sileshi et al. (2014), Tanveer et al. (2017), Castle et al. (2021)
Scattered agroforestry trees	A technology where agroforestry trees are dispersed in crop and grazing lands	Can adapt to different agroecological niches depending on the tree species and crop combination	Food, feeds and fodder provision, biomass source	Soil properties and fertility improvement, shade provision, microclimate control, biodiversity habitat and conservation, nutrients cycling	Low costs of tree management, income generation from different tree products	Competition between crops and trees for water and nutrients	Kelso and Jacobson, (2011), Tengnas (1994), Castle et al. (2021)
Terracing agroforestry technology	A technology of establishing trees on terraces along sloppy and hilly lands	Mostly applicable in sloppy highlands and hilly areas	Food, fodder, fuelwood production	soil and water conservation, soil fertility, erosion control, sedimentation control downstream	Increased farm yields and profitability	High costs of establishing (and maintaining) terraces in some landscapes	Kiptot & Franzel (2021), Onoja et al. (2019)
Silvopastoral/fodder agroforestry	Agroforestry technology that combines trees, shrubs, and forages with livestock production	Common in pasturelands and ranching systems, largely in the arid and semi-arid areas	Fodder, forage and food provision, fuelwood etc	Conserving biodiversity, carbon sinking, controlling soil erosion, shade provision, microclimate regulation	Income diversification	Potential introduction of invasive species	Balehegn, (2017), Silva-Galicia et al. (2020), Lemes et al. (2021)
Coffee agroforestry	Coffee production technology under different tree shading such as semi-forest, small-scale or large-scale coffee	Mostly in the East African highlands	Coffee production, food and fuelwood provision, timber, and non-timber products	Biodiversity conservation, soil fertility, microclimate regulation, shade provision	Income generation, employment creation	Deforestation for coffee expansion	Getachew (2013), Bucagu (2013), Jemal and Callo-Concha (2017), Duguma et al. (2021)
Fertility trees/shrubs systems	Development of tree and shrub in farmlands (for example *Glyricidia/Cassia siamea)* to enhance soil fertility through nitrogen-fixing to bring nutrients closer to the soil surface	multiple agroecological zones, especially those with degraded soils	Food, fodder, and fuelwood production	Soil fertility, nitrogen-fixing	Increased farm yield resulting in farm profitability		Teklehaimanot, (2004), Carsan et al. (2014), Cyamweshi et al. (2021)
Improved tree fallows technology	A technology of planting legume trees and crops in rotation arrangement within the farmland	Different agroecological niches depending on trees-crops arrangement	Food, fodder, timber and non-timber products, biomass provision	Improved land productivity, soil fertility, soil carbon sequestration	Rural income diversification	Crop-trees competition for nutrients, water, and other resources	Onoja et al. (2019), Kaczan et al. (2013)
Home gardens	A small-scale production system mainly involving fruits, herbs, vegetables and sometimes animals located near or around the homestead for domestic consumption.	Different niches varying with crop and animal types	Food, fodder, and feeds production	Erosion control, shade	Improved health, income diversification		Steppler and Nair (1987); Depenbusch et al. (2021).

### 4.2 Yearly distribution of studies

**Figure 3:**
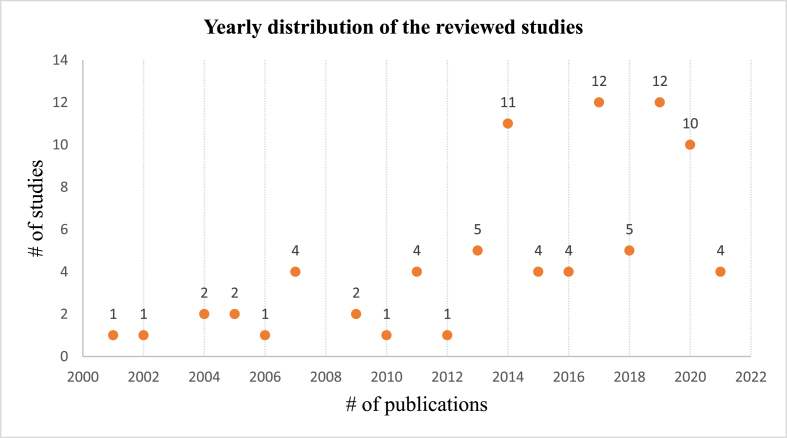
Yearly distribution of studies reviewed.

The studies reviewed ranged from the year 2001–2021, with the numbers ranging from 1 to 12 as presented in Figure 3. Notably, the selected studies are the ones that met the inclusion and exclusion criteria, thus there were more studies in agroforestry during the study years but were excluded for further analysis. The overall trend suggests an increasing number of agroforestry studies in the last two decades, resulting from factors like increased community awareness of agroforestry adoption and its benefits, government interventions through policies, institutional support, and extension services, as well as the development of markets and value chains to enhance the marketability of related goods and services. However, the levels of adoption generally remain low with a wide variance of agroforestry adoption at both intra and inter-country levels.

### 4.3 Geographical distribution of the studies

The geographical scope of the studies ranged from national to continental scales (some studies using SSA and continents outside Africa were included in this category) as presented in Table 3. Notably, several studies, especially conceptual ones, were cutting across different geographical scales, while primary data studies were primarily confined to one nation or locality. The study further explored how agroforestry policies and strategies are enshrined in the national frameworks of selected countries, particularly the National Adaptation Plans (NAPs), Nationally Determined Contributions (NDCs), and National Adaptation Programmes of Actions (NAPAs). In agreement with Bernard et al. (2019), there remains a huge gap in mainstreaming and integrating agroforestry in national and sectoral policies across Africa, thus the need for national strategies, policies, and action plans to guide national agroforestry implementation. Countries like Rwanda and South Africa clearly have agroforestry development strategies and action plans, with many other plans and strategies being developed by different countries across SSA.

**Table 3:** Geographical distribution of the studies reviewed.

Country/region	No. of publications	Context of agroforestry integration in the NDCs	Context of agroforestry integration in the NAPs/NAPAs
African region	30	-	-
Ethiopia	16	***Mitigation & adaptation*** (integrating climate change adaptation and expansion of agroforestry)	***Agriculture*** (Legume-based Agroforestry, home-gardens, on-farm and homestead forestry)
Cameroon	10	***Agriculture, forestry, and other land uses*** (including agropastoral)	***Agriculture*** (including agro-sylvo-pastoral and promoting on-farm trees)
Kenya	7	***Environment*** (plant 350,000 agroforestry trees in farmlands)	***Agriculture*** (Develop and up-scale specific adaptation actions)
Nigeria	6	***Mitigation & adaptation*** (emissions reduction, and nature-based solutions to climate change).	***Indigenous practices and knowledge***
Malawi	3	***Forestry and land use***—farm and forests resilience and restoration, and other co-benefits	***Community resilience/Sustainable livelihood***
Rwanda	3	***Land and forestry*** (livelihood and ecosystems co-benefits)	***Adaptation*** (e.g., agropastoral activities, developing agro-sylvo-pastoral systems)
South Africa	3	None	None
Tanzania	3	None	None
Zambia	3	***Forestry and Land Use*** (agroforestry for climate change mitigation)	***Sustainable livelihoods and agricultural activities***
Côte d'Ivoire	2	***Agriculture and forestry*** (including agropastoral and forests restoration)	None
Ghana	2	None	None
Liberia	2	***Agriculture and forestry*** (promote low-carbon agriculture practices and enhanced forest carbon stocks)	None
Sahel region	2	-	-
Uganda	2	None	***Agriculture*** (soil and water conservation trenches, fruit trees, woody species, fodder trees)
Burkina Faso	1	***Agriculture, Forestry and Other Land Uses*** (including agro-pastoral, emissions reduction)	***Food and fodder security*** (including Agro-sylvo-pastoral systems, fodder, and food production)
Comoros	1	***Agriculture and Livestock*** (agro-pastoral systems)	***Forestry*** (restoration of basin slopes)
Mali	1	***Agriculture and livestock*** (agro-pastoral development)	***Forestry and agriculture*** (reforestation, enhanced agro-sylvo-pastoral systems)
Morocco	1	None	None
Niger	1	***Agriculture***(promotion of agro-silvo-pastoral and agroforestry)	***Food and fodder security* (**agro-sylvo-pastoral development and food security)
Senegal	1	***Agriculture*** (sustainable land management; integrated agriculture- production & livestock systems)	***Adaptation and capacity development*** (including woodlots and fodder/pasture for livestock)
**Total**	**100**		

### 4.4 Production system and type of data used

The production systems were categorized into scientific (57%), indigenous (16%) or both systems (27%). The categories were based on the review of the studies to ascertain whether they employed a scientific research approach or ancient production systems. Farmers generally develop indigenous technologies to meet their plot or farm-scale needs without investment in production research. In contrast, scientific technologies are developed at the farm or landscape level using techno-scientific research and extension services (Foresta et al., 2000). The data type was categorized into field data or primary research (57%), conceptual (36%) and review (7%).

### 4.5 Type of studies

**Figure 4:**
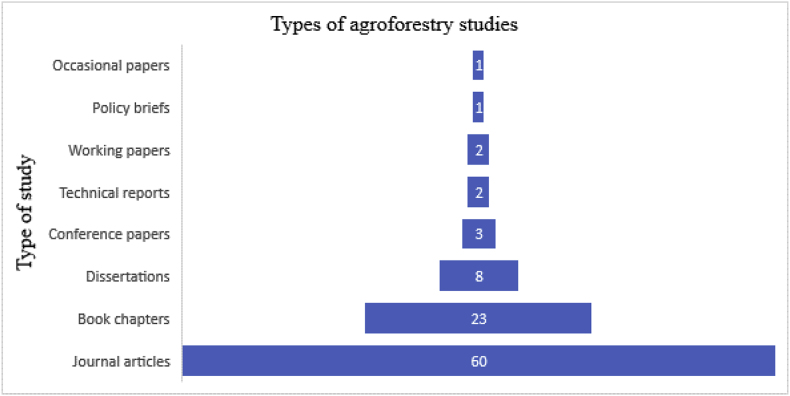
Types of studies reviewed.

The reviewed studies fell under different categories. The majority were peer-reviewed journal articles (60%), followed by book chapters at 23%. Other types included dissertations, conference papers, technical reports, occasional papers, and policy briefs as summarized in Figure 4.

### 4.6 Ecosystem services generated by different agroforestry technologies

The reason behind developing different agroforestry technologies is the generation of different ecosystem services to benefit people and ecosystems. Whereas the objective of the study is not to give the economic value of the benefits associated with agroforestry systems largely due to inadequate data, there are some indicative figures for different services as discussed in the sections below. Using 17 ecosystem services and 16 biomes, Constanza et al. (1997) calculated the economic value of global ecosystem services at $33 trillion annually, almost twice the total global gross national product. The study employed the Millennium Ecosystem Assessment (2005) categorization of ecosystem services (provisioning, support, regulatory, and cultural) to establish various ecosystem services derived from agroforestry technologies. This review found that most agroforestry technologies generate more than one category and type of ecosystem services in line with Muthee et al. (2018), who argue that agroforestry is multifunctioning and multiple beneficial in supporting livelihoods and ecological functioning.

#### 4.6.1 Provisioning services

**Figure 5:**
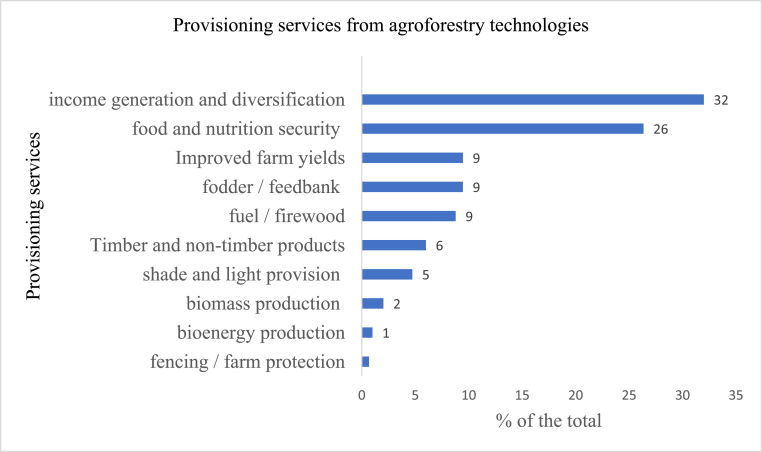
Provisioning services generated by agroforestry technologies.

The dominant provisioning service provided by agroforestry technologies studied is income generation or diversification to support livelihoods, accounting for 31% of the provisioning services mentioned as presented in Figure 5. Income is the driving motivation behind households engaging in tree cropping (Fouladbash and Currie, 2015). Different studies captured this service differently, including income generation, foreign exchange earnings, increased farm income, diversification of income sources, improved farm-level income, increased net income, poverty reduction, rural income diversification, increased profitability, rural business development, supplementing household income, revenues diversification and improved livelihoods [see for example: Leakey et al., 2005; Kerr, 2007; Fouladbash, 2013; Zira et al., 2016; Kassie and Yildiz, 2016]. Besides income generation, agroforestry promotes food security by providing food (fruits, nuts, vegetables etc) and enhancing food security (26% of studies reviewed). Various studies establish that agroforestry in Africa is primarily propelled by the need to increase food and nutritional security for practitioners [see for example: Jamnadass et al. (2011), Mbow et al. (2014), Jemal and Callo-Concha (2017), and Bright (2017). Agroforestry is a climate-smart agricultural technology with multiple livelihoods and ecosystem benefits. Manaye et al. (2020) estimate that agroforestry contributes to about 39.5% of the livelihood products. Other provisional services provided by agroforestry technologies include providing fodder and feeds, fuel and firewood, timber and non-timber products, and bioenergy and biomass production. The economic value of provisioning services varies with specific products, locations, niches, valuation methods, among other factors. To illustrate, El Tahir and Vishwanath (2015) used the net direct-use value to estimate the economic value of different non-timber products such as gum arabic and fodder in Eastern Sudan at USD 1,335,636.36 per household per annum. Using the return on investment and avoidance costs valuation approaches in Kelka Forest, Mali, Sidibé et al. (2014) estimated that restoration using reforestation and agroforestry gives a return of between 6% and 13% at local and global scales respectively in the long-term. These pointers are critical in estimating the value of provisioning services associated with agroforestry systems.

#### 4.6.2 Regulatory services

**Figure 6:**
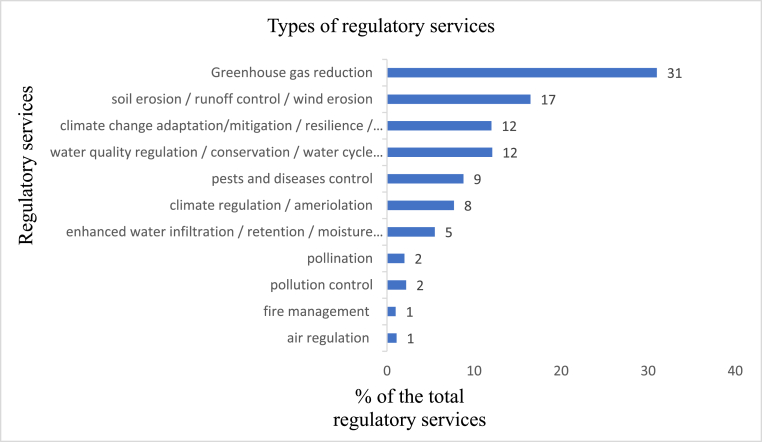
Regulatory services generated by different agroforestry technologies.

As Muthee et al. (2022) note, regulatory services aim to moderate ecosystem functioning. Carbon sequestration was noted as the dominant regulatory ecosystem service generated from agroforestry technologies at 31% of the mentioned regulatory services as summarized in Figure 6. The studies captured this role differently, including carbon sequestration, carbon storage, carbon sinking, greenhouse gases emissions reduction, methane reduction, aboveground tree carbon storage and increased carbon stock [see for example: Nair et al., 2009; Luedeling et al., 2014; Cyamweshi et al., 2021]. A study by Balasubramanian (2019) estimated the economic value of global regulating ecosystem services at US$29.085 trillion for 2015, with climate and water regulation services taking the lead. Agroforestry technologies also provide a critical regulatory service of erosion control, which was cited by 16% of the total regulatory services. This service was captured differently by the studies, including soil erosion prevention/control, surface water runoff control, wind erosion regulation/control, and reduced soil loss [see for example: Jose, 2009; Kiptot and Franzel, 2011; Kaczan et al., 2013; Andres et al., 2016; and Cyamweshi et al., 2021]. Further, adaptation and mitigation to climate change, and enhanced water and air quality regulation were identified as key regulatory services provided by agroforestry technologies at 12% of the regulatory services mentioned. Other regulatory services associated with agroforestry technologies development include fire management, pollution control, pollination, and enhanced water infiltration as established in Figure 6 below.

#### 4.6.3 Support services

**Figure 7:**
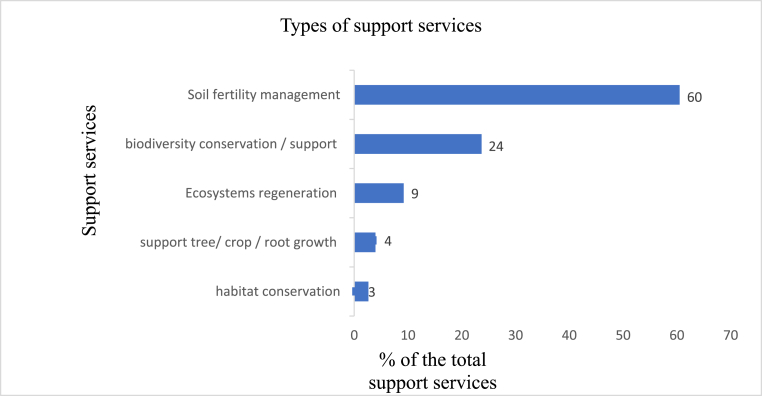
Support services generated by agroforestry technologies.

Supporting services sustain the production of the other ecosystem services. The reviewed studies revealed that soil formation and fertility management is the dominant support service generated by agroforestry technologies at 60% of the total supporting services mentioned (Figure 7). Various phrases were used to describe this service, including soil fertility management, improved soil organic matters, soil structure formation, soil enrichment, soil management, soil nutrients cycling, soil productivity, soil rehabilitation, soil replenishing, and soil quality restoration [see for example: Quisumbing et al., 2001; Kiptot and Franzel, 2011; Lasco et al., 2014; Ziyadi et al., 2019]. Soil formation and fertility management are essential for enhanced farm-level productivity. Agroforestry technologies also enhance biodiversity conservation and support. On-farm trees provide habitats to a wide range of species for mutual livelihoods and biodiversity benefits (Gockowski et al., 2010). Other essential support services associated with agroforestry technologies include habitat conservation, supporting crop and tree growth, and enhanced ecosystems health and regeneration.

#### 4.6.4 Cultural services

Different non-material benefits generated by ecosystems are classified as cultural services. The main cultural benefit associated with agroforestry is the traditional medicinal value which is dominant in the traditional agroforestry technologies. Other cultural benefits include cosmetology, aesthetic value, and cultural preservation especially for the indigenous communities (Sonwa et al., 2002; Teklehaimanot, 2004; Ziyadi et al., 2019; Silva-Galicia et al., 2020). However, cultural services remain the most understudied and undervalued amongst the studies reviewed, thus requiring more future studies. Kubiszewski et al. (2022) give some insights on choosing the right methodology(ies) for ecosystem services valuation, considering resources availability, scope, and desired precision of the valuation study.

## 5 Tradeoffs associated with agroforestry technologies

Despite numerous livelihood and ecosystem benefits associated with agroforestry technologies, it was established that there also exist various tradeoffs as described in Table 4. It is noteworthy that tradeoffs are context-specific depending on the agroecological niche and particular agroforestry technologies developed. To illustrate, establishing the right agroforestry technologies in the drylands may increase crop yields due to increased soil fertility and reduced soil erosion, while establishing the same in moist lands may increase the canopy cover leading to low productivity for the below crops. Introducing tree-based systems to farmlands could reduce crop productivity, which accounted for 23% of the total mentioned tradeoffs (including Fouladbash, 2013; Vaast and Somarriba, 2014; Kuyah et al., 2019; Sida et al., 2020). Conversion of croplands to agroforestry was the main driving factor, with some studies linking agroforestry to reduced farm profitability and food insecurity. The reviewed studies established the need for more research on the tree-crops combination with minimal tradeoffs and maximum benefits to both livelihoods and ecosystems. This could also be complemented by fertilizing and manuring the land to ensure farm productivity across the year. Twenty per cent (20%) of the mentioned tradeoffs were associated with tree-crops competition for water, light, and available nutrients (Kerr, 2007; Fouladbash and Currie, 2015; Sida et al., 2020). High deforestation, natural forests conversion to man-made forests, and the introduction of exotic species at the expense of indigenous ones accounted for 13% of the total tradeoffs (Quisumbing et al., 2001; Saj et al., 2017). Other tradeoffs established in the studies included excessing shading that may affect the growth of underground crops, loss of indigenous species due to the introduction of quick maturing exotic trees, and the emergence of pests and diseases.

**Table 4:** Tradeoffs associated with agroforestry technologies development.

Description of tradeoffs	no. of mentions	% of total tradeoffs
Reduced net carbon sequestration	1	3
Reduced agroecological resilience	1	3
Food insecurity as cereal lands are converted for tree growth	1	3
Loss of soil fertility, soil acidification	2	5
Introduction of invasive/alien species	2	5
Increased pests and diseases in croplands	3	8
Loss of indigenous tree genetic diversity	4	11
Excessive shade affecting undergrowth crops	4	11
Natural forests conversion	5	13
Reduced farm crop productivity	7	18
Tree-crop competition for water, fertile soils, etc	8	20
**Total**	**38**	**100**

Reducing tradeoffs related to agroforestry technologies require multidisciplinary approaches to meet related goods and service with minimal negative social and environmental impacts (Vaast and Somarriba, 2014). These include adopting the right agroforestry technology and growing the right high-yielding tree species at the right agroecological niches, right season, for the right purpose.

## 6 Barriers to the agroforestry technologies development

Developing effective and sustainable agroforestry technologies is threatened by a myriad of challenges. The dominant barriers cited by the reviewed studies were insecure land tenure systems and inadequate research and development on agroforestry technologies, each accounting for 20% of mentioned barriers as presented in Table 5. Studies like Fouladbash (2013), Kaczan et al. (2013), Lasco et al. (2014), Fouladbash and Currie (2015), and Partey et al. (2017) concur that land tenure systems, land rights, and equitable access to land are among the major impediments to successful agroforestry technologies development in SSA region.

**Table 5:** Barriers associated with agroforestry technologies development.

Description of barriers to the agroforestry technologies development	# of mentions	% of the total
Pests and diseases	1	2
Methodological difficulties in carbon sequestration assessment	1	2
Inadequate youth participation	1	2
Inadequate supply of farm inputs (seeds, seedlings)	1	2
Livestock intrusion/transhumance	1	2
Low institutional and extension support	2	5
Environmental barriers	3	7
High costs of AF technologies development	3	7
Inadequate technologies to measure AF returns	3	7
Market price fluctuation may affect farm profitability	4	10
Gender inequality in agroforestry participation	6	14
Inadequate research and development on AF technologies	8	20
Insecure land tenure systems	8	20
**Total**	**42**	**100**

Issues around inadequate knowledge, awareness and information, research on cross-cutting agroforestry benefits, matching the right species with the right ecological context and the right tree-crop combination were highly cited. Other barriers included difficulties in measuring agroforestry benefits to landscapes and livelihoods and inadequate access to contemporary agroforestry and development across the SSA region (see for example: Kelso and Jacobson, 2011; Partey et al., 2017).

Gender inequality is also cited as a major impediment to sustainable agroforestry technologies development in this region (Kiptot and Franzel, 2011). In particular, women are disadvantaged when accessing land, making decisions on which species to plant, and realizing full benefits associated with agroforestry-related products and services as Gockowski et al. (2010) assert. Gender inequality is cited as inadequate gender considerations, unequal men/women participation, inadequate women participation, and gender disparities (Sahilu, 2017). Other major factors cited by the studies include market fluctuation and inadequate value chain development in agroforestry, high costs associated with developing agroforestry technologies, livestock intrusion and transhumance, and pests and diseases.

## 7 Mechanisms and pathways toward reducing barriers and tradeoffs associated with agroforestry technologies adoption

There are various pathways proposed by the studies on increasing agroforestry systems adoption and different associated benefits as highlighted in Table 6. One of the barriers identified in the studies was the inadequate supply of farm inputs, including seeds, seedlings, fertilizers, and tree protection guards. To address this challenge, Partey et al. (2017) propose developing seed orchards and nurseries for sustainable seeds and seedlings supply and income generation. Further, studies including Kassie and Yildiz (2016) and Onoja et al. (2019) suggest improved access to farm inputs such as fertilizer, planting materials, agrochemical, tools, and machinery required for tree systems development. These inputs have significantly increased costs associated with agroforestry technologies development, contributing to slow adoption levels despite their potential to deliver multiple benefits in the SSA region.

**Table 6:** A look at the barriers to agroforestry technologies adoption, potential redress pathways and associated benefits.

Barriers of agroforestry adoption	Pathway to addressing the barriers	Associated benefits
Inadequate supply of farm inputs	Development of household and community seed orchards and nurseries	Income generation, sustainable supply of seeds and seedlings
	Improved access to farm inputs	Reduced costs of AF development, increased AF adoption
Inadequate institutional and policy environment	Enactment of contextualised national and sectoral policies to promote AF adoption	Conducive environment for AF adoption and its associated benefits
	Extension services	Information access, increased awareness, enhanced adoption, AF diversification
Insufficient rewards and incentives for AF related goods	Enhance business support systems	Income generation, value chains development, markets development

The right institutional and policy environment for agroforestry development is also an essential pathway toward increasing agroforestry adoption. Studies such as Partey et al. (2017) propose policies to address market failure and incentivize on-farm tree growing, Kassie and Yildiz (2016) suggest policies that motivate agroforestry adoption and income diversification, Amare et al. (2019) support policies on equitable access to land resources, while Onoja et al. (2019) suggest policies on climate-smart agriculture adoption. In essence, policies should be contextualized to meet the local needs and demands for sustainable agroforestry adoption. The study has looked at how different strategies and frameworks, including the NDCs, NAPAs and NAPs have integrated agroforestry in different sectors, but more needs to be done to ensure the implementation of these suggestions.

From the institutional perspective, the studies suggested more investment in extension and business support services. Extension services are crucial in increasing knowledge, awareness and information on cross-cutting benefits associated with agroforestry technologies development in the local communities. Tadesse et al*.* (2021) suggest that extension officers can aid the communities in matching trees and crops in ideal landscapes to enhance the uptake and scaling up of agroforestry technologies. Business support services are essential in increasing and diversifying farm-level income generated by agroforestry goods and services. These include the development of value chains, rural markets and infrastructures, and improved access to credit services to support farmers involved in agroforestry technologies (Bullock et al., 2013; Zira et al., 2016). Value addition of agroforestry goods and services remains low in most of the studies. If well developed, value addition can directly increase farmers' income, resulting in higher adoption of agroforestry technologies at the farm level. These may include processing and packaging juice from fruit orchards, honey and wax production and packaging from apicultural products, and non-timber products such as herbal medicines, wild fruits, and handcraft items (Amare et al., 2019; Sheppard et al., 2020; Elagib and Al-Saidi, 2020). In so doing, agroforestry can have cross-cutting benefits both to the livelihoods and ecosystems.

## 8 Concluding thoughts

Agroforestry remains one of the old concepts that is attracting new research and development interests globally due to its potential to promote ecosystems restoration and improve rural livelihoods especially in the Sub-Saharan Africa. The study has identified three distinct phases of from traditional agroforestry practices, agroforestry concept development and institutionalization, to agroforestry research and policy mainstreaming, each with distinct features. The review suggested that the agroforestry is beneficial to the practitioners, though with potential tradeoffs that should be addressed for it to be sustainable. The review is however limited to only those studies that met the inclusion and exclusion criteria, implying that some information related to agroforestry across the sub-Saharan Africa might have been missed out. As such it recommends more country specific reviews of the existing studies and practices to understand different social, environmental, and economic dynamics of agroforestry. Afterall, agroforestry is context specific and is practiced depending on factors such as awareness level, resources available, prevailing sociocultural aspects, desired outcomes, and existing biophysical and agroecological conditions.

## Declarations

### Author contribution statement

Kennedy Muthee, Lalisa Duguma, Christine Majale, Monicah Mucheru-Muna, Priscilla Wainaina, Peter Minang: Conceived and designed the analysis; Analyzed and interpreted the data; Contributed analysis tools or data; Wrote the paper.

### Funding statement

This research did not receive any specific grant from funding agencies in the public, commercial, or not-for-profit sectors.

### Data availability statement

Data included in article/supplementary material/referenced in article.

### Declaration of interest’s statement

The authors declare no conflict of interest.

### Additional information

No additional information is available for this paper.

## Appendix A Supplementary data

Appraisal of selected agroforestry studies_database Appraisal of selected agroforestry studies_database

## Footnotes

1 Publish or Perish on Microsoft Windows (harzing.com).

2 PRISMA (prisma-statement.org).

## References

[bib1] Akamani K., Holzmueller E.J., Dagar J., Tewari V. (2017). Agroforestry.

[bib2] Amare D., Wondie M., Mekuria W. (2019). Agroforestry of smallholder farmers in Ethiopia: practices and benefits. Small-Scale Forest..

[bib3] Andersson L. (2018). Achieving the Global Goals through Agroforestry.

[bib4] Andres C., Comoé H., Beerli A., Schneider M., Rist S., Jacobi J., Lichtfouse E. (2016). Sustainable Agriculture Reviews. Sustainable Agriculture Reviews 19.

[bib5] Asase A., Tetteh D.A. (2010). The role of complex agroforestry systems in the conservation of forest tree diversity and structure in Southeastern Ghana. Agrofor. Syst..

[bib6] Balasubramanian M. (2019). Economic value of regulating ecosystem services: a comprehensive at the global level review. Environ. Monit. Assess..

[bib7] Balehegn M., Leal Filho W., Belay S., Kalangu J., Menas W., Munishi P., Musiyiwa K. (2017). Climate Change Adaptation in Africa. Climate Change Management Series.

[bib8] Bernard F., Bourne M., Garrity D., Neely C., Chomba S. (2019).

[bib9] Bjornlund V., Bjornlund H., Rooyen A. (2020). Why agricultural production in sub-Saharan Africa remains low compared to the rest of the world—a historical perspective. Int. J. Water Resour. Dev..

[bib10] Bright M.B.H. (2017). The Role of Shrub Agroforestry Systems in Increasing Food Security for the West African Sahel [Doctoral Dissertation. http://rave.ohiolink.edu/etdc/view?acc_num=osu1503071499488486.

[bib11] Bucagu C. (2013).

[bib12] Buck L. (1981). Kenya National Seminar on Agroforestry Proceedings. 12-22 November 1980.

[bib13] Bullock R., Mithöfer D., Vihemäki H. (2013). Sustainable agricultural intensification: the role of cardamon agroforestry in the East Usambaras, Tanzania. Int. J. Agric. Sustain..

[bib14] Carsan S., Stroebel A., Dawson I., Kindt R., Mbow C., Mowo J., Jamnadass R. (2014). Can agroforestry option values improve the functioning of drivers of agricultural intensification in Africa?. Curr. Opin. Environ. Sustain..

[bib15] Castle S.E., Miller D.C., Ordonez P.J., Baylis K., Hughes K. (2021). The impacts of agroforestry interventions on agricultural productivity, ecosystem services, and human well-being in low- and middle-income countries: a systematic review. Campbell Syst. Rev..

[bib16] Conklin H.C. (1957). Forestry Development Paper No. 12.

[bib17] Costanza R., d'Arge R., De Groot R., Farber S., Grasso M., Hannon B., Limburg K., Naeem S., O'neill R.V., Paruelo J., Raskin R.G. (1997). The value of the world's ecosystem services and natural capital. Nature.

[bib18] Cyamweshi A.R., Kuyah S., Mukuralinda A., Muthuri C. (2021). Potential of *Alnus acuminata* based agroforestry for carbon sequestration and other ecosystem services in Rwanda. Agrofor. Syst..

[bib19] Delaquis E., De Haan S., Wyckhuys K. (2017). On-farm diversity offsets environmentalpressures in tropical agroecosystems: a synthetic review for cassava-based systems. Agric. Ecosyst. Environ..

[bib20] Depenbusch L., Schreinemachers P., Roothaert R., Namazzi S., Onyango C., Bongole S., Mutebi J. (2021). Impact of home garden interventions in East Africa: results of three randomized controlled trials. Food Policy.

[bib21] Duguma L.A., Nzyoka J., Minang P.A., Bernard F. (2017). ICRAF Policy Brief No. 34.

[bib23] Duguma L.A., van Noordwijk M., Minang P.A., Muthee K. (2021). COVID-19 pandemic and agroecosystem resilience: early insights for building better futures. Sustainability.

[bib24] Duguma L.A., Muthee K., Carsan S., Muriuki J., Bulitta B.J., Ayana A.N., Kibugi R.M., Suleman K.K., Minang P.A., Duguma L.A., van Noordwijk M. (2021). Tree Commodities and Resilient Green Economies in Africa.

[bib25] Edwards-Callaway L.N., Cramer M.C., Cadaret C.N., Bigler E.J., Engle T.E., Wagner J.J., Clark D.L. (2021). Impacts of shade on cattle well-being in the beef supply chain. J. Anim. Sci..

[bib26] El Tahir B., Vishwanath A. (2015). Estimation of economic value of agroforestry systems at the local scale in eastern Sudan. J. Geosci. Environ. Protect..

[bib27] Elagib N.A., Al-Saidi M. (2020). Balancing the benefits from the water–energy–land–food nexus through agroforestry in the Sahel. Sci. Total Environ..

[bib29] Faße A., Winter E., Grote U. (2014). Bioenergy and rural development: the role of agroforestry in a Tanzanian village economy. Ecol. Econ..

[bib30] Foresta H., Michon G., Kusworo A. (2000).

[bib31] Fouladbash L. (2013).

[bib32] Fouladbash L., Currie W. (2015). Agroforestry in Liberia: household practices, perceptions and livelihood benefits. Agrofor. Syst..

[bib33] Franzel S., Cooper P., Denning G.L., Eade D. (2002). Development and Agroforestry: Scaling up the Impacts of Research.

[bib34] Gockowski J., Tchatat M., Dondjang J., Hietet G., Fouda T. (2010). An empirical analysis of biodiversity and economic returns to cocoa agroforestry in Southern Cameroon. J. Sustain. For..

[bib35] Hsiung W., Yang S., Tao Q., Sinclair F.L. (1995). Agroforestry: Science, Policy and Practice. Forestry Sciences.

[bib36] Huxley P.A. (1983). Plant Research and Agroforestry Proceedings of a Consultative Meeting in Nairobi, Kenya.

[bib37] Huxley P.A. (1999).

[bib38] Jamnadass R.H., Dawson I.K., Franzel S., Leakey R.R.B., Mithöfer D., Akinnifesi F.K., Tchoundjeu Z. (2011). Improving the livelihoods and nutrition in sub-Saharan Africa through the promotion of indigenous and exotic fruit production in stallholders’ agroforestry systems: a review. Int. For. Rev..

[bib39] Jemal O.M., Callo-Concha D. (2017). Southwestern Ethiopia ZEF Working Paper Series, No. 161.

[bib40] Jose S. (2009). Agroforestry for ecosystem services and environmental benefits: an overview. Agrofor. Syst..

[bib41] Kaczan D., Arslan A., Lipper L. (2013). ESA Working Paper No. 13-07.

[bib42] Kassie G.W., Yildiz F. (2016). Agroforestry and land productivity: evidence from rural Ethiopia. Cogent Food & Agricul..

[bib43] Kelso A., Jacobson M. (2011). Community assessment of agroforestry opportunities in GaMothiba, South Africa. Agrofor. Syst..

[bib44] Kerr A.C. (2007).

[bib45] Kilewe A.M., Kealey K.M., Kebaara K.X. (1989).

[bib46] King K.F.S., Steppler H.A., Nair P.K.R. (1987). Agroforestry: A Decade of Development.

[bib47] Kiptot E., Franzel S. (2011).

[bib48] Kubiszewski I., Muthee K., Rasheed R., Costanza R., Suzuki M., Noel N., Schauer M. (2022). The cost of increasing precision for ecosystem services valuation studies. Ecol. Indicat..

[bib49] Kumar A., Oraon P., Singh B. (2020). Advances in Agricultural Sciences.

[bib50] Kuyah S., Whitney C.W. (2019). Agroforestry delivers a win-win solutions for ecosystem services in sub-Saharan Africa: a meta-analysis. Agron. Sustain. Dev..

[bib51] Lasco R.D., Delfino R.J., Espaldon M. (2014). Agroforestry systems: helping smallholders adapt to the climate risks while mitigating climate change. Wiley Interdiscipl. Rev.: Clim. Change.

[bib52] Leakey R.R.B., Tchoundjeu Z., Schreckenberg K., Shackleton S.E., Shackleton C.M. (2005). Agroforestry tree products (AFTPs): targeting poverty reduction and enhanced livelihoods. Int. J. Agroforest. Sustain..

[bib53] Luedeling E., Kindt R., Huth N., Koenig K. (2014). Agroforestry systems in a changing climate—challenges in projecting future performance. Curr. Opin. Environ. Sustain..

[bib54] Lundgren B. (1982). Introduction [editorial]. Agrofor. Syst..

[bib55] Malkamäki A., D’Amato D., Hogarth N., Kanninen M., Pirard R., Toppinen A., Zhou W. (2018). A systematic review of socioeconomic impacts of large-scale tree plantations worldwide. Global Environ. Change.

[bib56] Manaye A., Tesfamariam B., Tesfaye M., Worku A., Abriha H., Gufi Y., Mekonnen Z. (2020). Conference: Current Information and Technologies on the Environment and Forests, Adama, Ethiopia.

[bib57] Mbow C., van Noordwijk M., Luedeling E., Neufeldt H., Minang P., Kowero G. (2014). Agroforestry solutions to address food security and climate change challenges in Africa. Curr. Opin. Environ. Sustain..

[bib58] Menzies N. (1988). Three hundred years of Taungya: a sustainable system of forestry in south China. Hum. Ecol..

[bib59] Meybeck A., Gitz V., Wolf J., Wong T. (2020).

[bib60] Millennium Ecosystem Assessment (MEA) (2005).

[bib61] Monteith J.L., Ong C.K., Corlett J.E. (1991). Microclimatic interactions in agroforestry systems. For. Ecol. Manage..

[bib62] Muschler R., Pancel L., Köhl M. (2016). Tropical Forestry Handbook.

[bib63] Muthee K., Mbow C., Macharia G., Leal-Filho W., Leal Filho W., Belay S., Kalangu J., Menas W., Munishi P., Musiyiwa K. (2017). Climate Change Adaptation in Africa. Climate Change Management.

[bib64] Muthee K.W., Mbow C., Macharia G.M., Leal-Filho W. (2018). Ecosystem services in adaptation projects in West Africa. Int. J. Clim. Change Strateg. Manag..

[bib65] Muthee K., Duguma L., Nzyoka J., Minang P. (2021). Ecosystem-based adaptation practices as nature-based solution to promote water-energy-food nexus balance. Sustainability.

[bib66] Muthee K., Duguma L., Wainaina P., Minang P., Nzyoka J. (2022). A review of global policy mechanisms designed for tropical forests conservation and climate risks management. Front. For. Glob. Change.

[bib67] Nair P.K.R. (1993).

[bib68] Nair P.K.R., Kumar B.M., Nair V.D. (2009). Agroforestry as a strategy for carbon sequestration. J. Plant Nutr. Soil Sci..

[bib69] Niang I., Ruppel O.C., Abdrabo M.A., Essel A., Lennard C., Padgham J., Urquhart P. (2014). Climate Change 2014: Impacts, Adaptation, and Vulnerability.

[bib70] Onoja A.O., Agbomedarho J., Etela I., Ajie E.N., Castro P., Azul A., Leal Filho W., Azeiteiro U. (2019). Climate Change-Resilient Agriculture and Agroforestry. Climate Change Management.

[bib71] Partey S., Sarfo D., Frith O., Kwaku M., Thevathasan N. (2017). Potentials of bamboo-based agroforestry for sustainable development in sub-saharan Africa: a review. Agric. Res..

[bib72] Quisumbing A., Aidoo J.B., Payongayong E., Otsuka K., Place F., Place Frank M. (2001). Land Tenure and Natural Resource Management: A Comparative Study of Agrarian Communities in Asia and Africa. Keijiro Otsuka.

[bib73] Raintree J.B., Warner K. (1986). Agroforestry pathways for the intensification of shifting cultivation. Agrofor. Syst..

[bib74] Rosenstock T., Wilkes A., Jallo C., Namoi N., Bulusu M., Suber M., Mboi D., Mulia R., Simelton E., Richards M., Gurwick N., Wollenberg E. (2019).

[bib75] Sahilu M.G. (2017).

[bib76] Saj S., Jagoret P., Etoa L.E., Fonkeng E.E., Tarla J.N., Nieboukaho J.E., Sakouma K.M. (2017). Lessons learned from the long-term analysis of cacao yield and stand structure in central Cameroonian agroforestry systems. Agric. Syst..

[bib77] Samra J.S., Charan S.S. (2000). Silvipasture systems for soil, water and nutrient conservation on degraded lands of Shivalik foothills (subtropical northern India). Indian J. Soil Conserv..

[bib78] Sheppard J.P., Bohn Reckziegel R., Borrass L., Chirwa P.W., Cuaranhua C.J., Hassler S.K. (2020). Agroforestry: an appropriate and sustainable response to a changing climate in southern Africa?. Sustainability.

[bib79] Sida T.S., Baudron F., Ndoli A. (2020). Should fertilizer recommendations be adapted to parkland agroforestry systems? Case studies from Ethiopia and Rwanda. Plant Soil.

[bib80] Sidibé Y., Myint M., Westerberg V. (2014). Report for the Economics of Land Degradation Initiative.

[bib81] Sileshi G.W., Mafongoya P.L., Akinnifesi F.K., Phiri E., Chirwa P., Beedy T., Makumba W., Nyamadzawo G., Njoloma J., Wuta M., Nyamugafata P., Jiri O., Van Alfen Neal (2014). In-Chief. Encyclopedia of Agriculture and Food Systems, 1.

[bib82] Silva-Galicia A., Valencia V., Arroyo-Rodríguez V., Moreno-Calles A., Ceccon E. (2020).

[bib83] Singh A., Tanwar S.P.S., Maghwal P.R., Saxena A., Kumar M. (2018). Assessing productivity and profitability of a rejuvenated ber (Ziziphus mauritiana) based agri-horti system under arid rainfed conditions. Indian J. Agric. Sci..

[bib84] Smith J. (2010).

[bib85] Sollen-Norrlin M., Bhim B.G., Naomi L.J.R. (2020). Agroforestry benefits and challenges for adoption in Europe and beyond. Sustainability.

[bib86] Sonwa D.J., Okafor J.C., Mpungi Buyungu P., Weise S.F., Tchatat M., Adesina-Nkongmeneck A.B., Ndoye O., Endamana D. (2002). Dacryodes edulis, A neglected non-timber forest species for the agroforestry systems of west and central Africa. For. Trees Liveli..

[bib87] Steppler H., Nair P.K.R. (1987). Agroforestry a Decade of Development.

[bib88] Tanveer M., Anjum S.A., Hussain S., Cerdà A., Ashraf U. (2017). Relay cropping as a sustainable approach: problems and opportunities for sustainable crop production. Environ. Sci. Pollut. Res. Int..

[bib89] Teklehaimanot Z. (2004). Exploiting the potentials of indigenous agroforestry trees: parkia biglobosa and Viltellaria paradoxa in sub-Sahara Africa. Agrofor. Syst..

[bib90] Tengnas B. (1994).

[bib91] UNU (1984).

[bib92] Vaast P., Somarriba E. (2014). Tradeoffs between crop intensification and ecosystem services: the role of agroforestry in cocoa cultivation. Agrofor. Syst..

[bib93] van Noordwijk M. (2019). Sustainable Development through Trees on Farms: Agroforestry in its Fifth Decade.

[bib94] Waldron A., Garrity D., Malhi Y., Girardin C., Miller D.C., Seddon N. (2017). Agroforestry can enhance food security while meeting other Sustainable Development Goals. Trop. Conserv. Sci..

[bib95] Zinngrebe Y., Borasino E., Chiputwa B. (2020). Agroforestry governance for operationalizing the landscape approach: connecting conservation and farming actors. Sustain. Sci..

[bib96] Zira B., Arifalo E., Apkan M., Madugu A. (2016). Socio-economic implication of agroforestry practices in southern kaduna, Nigeria. J. For. Sci. Env..

[bib97] Ziyadi M., Dahbi A., Aitlhaj A., El Ouahrani A., El Ouahidi A., Achtak H., Castro P., Azul A., Leal Filho W., Azeiteiro U. (2019). Climate Change-Resilient Agriculture and Agroforestry. Climate Change Management.

